# Sex Determination: Why So Many Ways of Doing It?

**DOI:** 10.1371/journal.pbio.1001899

**Published:** 2014-07-01

**Authors:** Doris Bachtrog, Judith E. Mank, Catherine L. Peichel, Mark Kirkpatrick, Sarah P. Otto, Tia-Lynn Ashman, Matthew W. Hahn, Jun Kitano, Itay Mayrose, Ray Ming, Nicolas Perrin, Laura Ross, Nicole Valenzuela, Jana C. Vamosi, The Tree of Sex Consortium

**Affiliations:** 1 University of California, Berkeley, Department of Integrative Biology, Berkeley, California, United States of America; 2 University College London, Department of Genetics, Evolution and Environment, London, United Kingdom; 3 Fred Hutchinson Cancer Research Center, Divisions of Human Biology and Basic Sciences, Seattle, Washington, United States of America; 4 University of Texas, Department of Integrative Biology, Austin, Texas, United States of America; 5 University of British Columbia, Department of Zoology, Vancouver, British Columbia, Canada; 6 University of Pittsburgh, Department of Biological Sciences, Pittsburgh, Pennsylvania, United States of America; 7 Indiana University, Department of Biology, Bloomington Indiana, United States of America; 8 National Institute of Genetics, Ecological Genetics Laboratory, Mishima, Shizuoka, Japan; 9 Tel Aviv University, Department of Molecular Biology and Ecology of Plants, Tel Aviv, Israel; 10 University of Illinois, Department of Plant Biology, Urbana-Champaign, Illinois, United States of America; 11 University of Lausanne, Department of Ecology and Evolution, Lausanne, Switzerland; 12 University of Oxford, Department of Zoology, Oxford, United Kingdom; 13 Iowa State University, Department of Ecology, Evolution and Organismal Biology, Ames, Iowa, United States of America; 14 University of Calgary, Department of Biological Sciences, Calgary, Alberta, Canada

## Abstract

Sexual reproduction is an ancient feature of life on earth, and the familiar X and Y chromosomes in humans and other model species have led to the impression that sex determination mechanisms are old and conserved. In fact, males and females are determined by diverse mechanisms that evolve rapidly in many taxa. Yet this diversity in primary sex-determining signals is coupled with conserved molecular pathways that trigger male or female development. Conflicting selection on different parts of the genome and on the two sexes may drive many of these transitions, but few systems with rapid turnover of sex determination mechanisms have been rigorously studied. Here we survey our current understanding of how and why sex determination evolves in animals and plants and identify important gaps in our knowledge that present exciting research opportunities to characterize the evolutionary forces and molecular pathways underlying the evolution of sex determination.

## Abstract 2

Sex is universal amongst most eukaryotes, yet a remarkable diversity of sex determining mechanisms exists. We review our current understanding of how and why sex determination evolves in animals and plants.

## Introduction

Sex—the mixing of genomes via meiosis and fusion of gametes—is nearly universal to eukaryotic life and encompasses a diverse array of systems and mechanisms [1]. One major role of sex is to bring together alleles carried by different individuals, revealing beneficial genetic variance that is otherwise hidden [2]. While many unicellular organisms produce gametes of equal size (isogamy, see Box 1), sexual reproduction in most multicellular organisms has led to the evolution of female and male gametes differing in size (anisogamy), and often to the evolution of two separate sexes. Even though the outcome of sex determination—whether an individual produces relatively few large ova or many small sperm—is strongly conserved, a bewildering number of underlying mechanisms can trigger development as either a male or female [3],[4].

**Box 1. From Mating Types to Sexes**

Meiotic sex likely has a single origin, which dates back to the origin of eukaryotes [144],[145]). While most eukaryotes display some form of meiotic sex, many lack differentiated male and female gametes—a situation referred to as isogamy. Even with isogamy, however, mating is often not random but requires that joining cells differ at a mating type (MAT) locus. Mating types might have evolved to orchestrate the developmental transition from the haploid to the diploid phase of the life cycle [146],[147]: *plus* and *minus* gametes express complementary transcription factors, encoded by different alleles at the MAT locus; these combine in the zygote into heterodimers that silence the genes expressed in the haploid phase and switch on the diploid program.

Isogamy permits a theoretically unlimited number of mating types; high numbers increase the probability that randomly mating partners display complementarity. Most basidiomycete fungi, for instance, present two independent MAT loci (and are therefore said to be tetrapolar, because a single meiosis can produce cells of four distinct mating types); each locus can be multiallelic, resulting in up to thousands of different mating types. Alternatively, a low probability of encountering complementary partners might have driven a transition to homothallism observed in many ascomycete fungi, which refers to a mating compatibility between genetically identical individuals. Homothallism evolved via genic capture: a single genome harbors complementary mating-type alleles, which are differentially expressed in *plus* and *minus* gametes. Mating-type switching in yeasts allows different cells from the same clone to express complementary mating types, and thus enter the diploid phase of their life cycle.

Anisogamy (small male and large female gametes) evolved independently in many eukaryotic lineages, including several different groups of protists (such as red algae, brown algae, apicomplexa, dinoflagellates, and ciliates; [148]), as well as most plants and animals. The transition towards anisogamy is thought to result from disruptive selection [1],[149],[150]: given opposing pressures to simultaneously maximize the number of gametes, their encounter rate, as well as the mass and ensuing survival of resulting zygotes, the fitness of both partners is often maximized when one interacting gamete is small and mobile, while its large and sessile partner provides the resources required for zygote development. Intermediate gametes do worse than small ones in terms of mobility and numbers, and worse than large ones in terms of provisioning. Such constraints largely explain why sexes (at the gametic level) are two and only two, and why anisogamy independently evolved in many lineages. At the molecular level, one route to anisogamy is by the incorporation of genes controlling gamete size into the MAT region [151]. Further extensions of the MAT region, possibly involving additional sex-antagonistic genes, led to the U and V chromosomes characterizing male and female gametophytes, as found, e.g., in mosses and liverworts [152].

Importantly, the evolution of anisogamy does not require the evolution of separate sexes, because hermaphrodites can produce both sperm and eggs. Similarly, several unicellular organisms that are anisogamous, such as apixomplexa and dinoflagellates, can make cells that produce sperm as well as cells that produce eggs. The evolution of completely separate sexes, where individuals cannot give rise to both sperm and egg descendants, is thought to be fairly derived and is found primarily among multicellular organisms with rare unicellular exceptions (e.g., the ciliate *Vorticella* [153] and several dioecious diatoms [154]).

**Figure 1:**
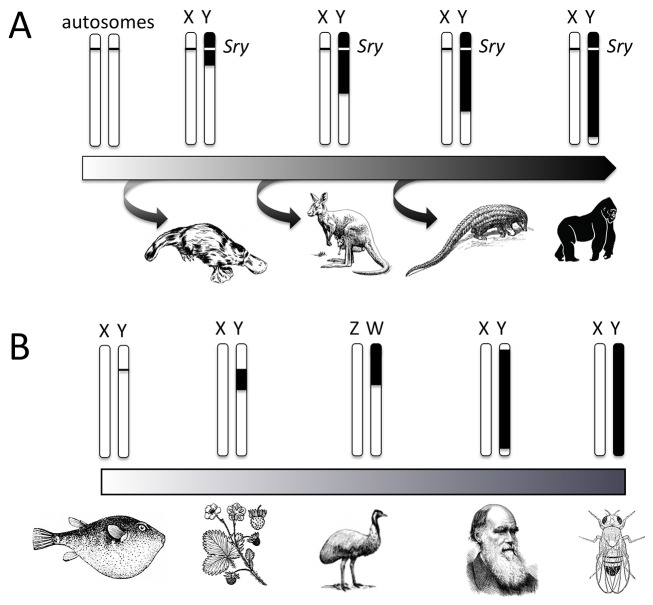
Sex chromosome differentiation. **A.** Reconstructed evolutionary path of sex chromosome differentiation in humans. Sex chromosomes originate from autosomes that acquired a sex-determining function (the *Sry* gene) after their split from monotremes. Suppression of recombination between the sex chromosomes, associated with degeneration of the non-recombining region of the Y chromosome, results in the morphological and genetic differentiation of sex chromosomes. Recombination suppression occurred in multiple episodes along the human X and Y chromosome, forming so-called evolutionary strata. The oldest stratum is shared between eutherian mammals and marsupials, while the youngest stratum of humans is primate-specific. **B.** The degree of sex chromosome differentiation ranges widely across species, spanning the entire spectrum of homomorphic to heteromorphic sex chromosomes, from a single sex-determining locus, as seen in pufferfish, a small differentiated region (strawberry and emu), most of the sex chromosomes apart from short recombining regions (humans), to the entire sex chromosome pair, as seen in *Drosophila*. Note that the sex chromosomes are not drawn to scale.

In humans, sex is determined by sex chromosomes (XX females, XY males). The X and Y chromosomes harbor dramatically different numbers and sets of genes (about 1,000 genes on the X and only a few dozen genes on the Y), yet they originated from ordinary autosomes during the early evolution of mammals (Figure 1). Restriction of recombination followed by gene loss on the Y has resulted in the morphological differentiation of sex chromosomes (for a review of the molecular and evolutionary processes involved in Y degeneration, see [4],[5]). The vast majority of genes on the sex chromosomes are not directly involved in sex determination, and development as a male or female depends on the presence of a single master sex-determining locus, the *Sry* gene, on the male-limited Y chromosome. Expression of *Sry* early in embryonic development initiates testis differentiation by activating male-specific developmental networks, while in its absence, ovaries develop. The first visible signs of sexual differentiation of the ovary and testis occur by the sixth week of gestation in humans [6], and sex hormones initiate further sexual differentiation in nongonadal tissues and organs [7]. When this developmental process goes awry, the effects can be catastrophic, causing everything from ambiguous external genitalia (which occurs in up to one in 4,500 infants) to sterility (which is more cryptic and difficult to diagnose but may be far more common).

Like humans and most mammals, other genetic model systems, such as *Drosophila melanogaster* flies and *Caenorhabditis elegans* nematodes, harbor sex chromosomes, and their commonalities have led to general assumptions about the conservation of sex determination mechanisms. However, these model organisms present a false impression of stability in how sex is determined, and their commonalities mask the diversity and turnover in sex determination mechanisms that is readily apparent when taking a broader taxonomic view. In this article, we address three common myths about sex determination and then deconstruct them based on a broad taxonomic survey of animals and plants.

## Myths of Sex Determination

### Myth 1: Sex is typically determined by X and Y chromosomes

Many biologists are habituated to thinking about sex determination through the familiar examples of mammals and *D. melanogaster*, and assume that sex determination by sex chromosomes is the norm, that males are XY and females are XX, and that sex chromosomes are a stable component of the genome. While biologists are generally aware of other modes of sex determination (such as female heterogamety in birds, temperature-dependent sex determination in reptiles, or development of males from unfertilized eggs in bees), these alternatives are often viewed as strange and aberrant [8].

### Myth 2: Sex is controlled by one master-switch gene

Sex determination in model species suggests that a master-switch gene (e.g. *Sry* in mammals, *Sxl* in *D. melanogaster*, and *xol-1* in *C. elegans*) acts as the main control element to trigger either male or female sexual development. Changes in the sex determination pathways across taxa are assumed to involve adding a new master-switch gene to this molecular pathway (as in some fly taxa; [9]), with little change to downstream elements of the sex determination pathway [10]. A few genes are thought to have the capacity to take on the role of sex determination genes, and these have been co-opted as master-switch genes independently in different lineages (for example, *dmrt1* in several vertebrates [11]–[14] and *tra* in insects [15]–[17]).

### Myth 3: Sex chromosome differentiation and degeneration is inevitable

Sex chromosomes originate from identical autosomes by acquiring a sex determination gene (for example, the origin of the *Sry* gene in mammals approximately 180 million years ago or *Sxl* in the *Drosophila* genus >60 million years ago). They are then thought to differentiate through an inevitable and irreversible process in which recombination between X and Y chromosomes is shut down and the Y degenerates (see Figure 1). Ultimately, Y chromosomes are fated to disappear entirely (“born to be destroyed,” [18]). Thus, sex chromosomes that are morphologically similar (homomorphic) must be evolutionarily young, and in time they too will degenerate.

## The Myths Deconstructed

These myths do not survive a survey of sex determination systems across the tree of life. To deconstruct these myths, we first provide background on the evolution of separate sexes. We then summarize the diversity of sex-determining mechanisms found among animals and plants and discuss the evolutionary forces that drive transitions among systems (Myth 1 revisited). This is followed by a summary of more recent findings on the underlying molecular genetics of sex determination (Myth 2 revisited) and a deconstruction of common misconceptions of sex chromosome evolution in humans and other species (Myth 3 revisited). We conclude with an outlook for future research that might improve our understanding of how and why sex determination evolves so rapidly in many animals and plants.

## The Evolution of Separate Sexes

While the evolution of anisogamy led to the evolution of male and female functions, the evolution of separate sexes is not inevitable across lineages. Indeed, most flowering plants (94%, [19]) have both male and female sex organs within a single individual and often within the same flower. By contrast, hermaphroditism is rare among animals considered as a whole (about 5% of all species), which is largely due to the absence of hermaphrodites in the species-rich insects, but it is common in many other animal taxa, including fish and many invertebrates (most snails, corals, trematodes, barnacles, and many echinoderms) [20]. Hermaphrodites can mate with each other and benefit from the advantages of sex by mixing their genomes, but when mates are difficult to find, hermaphrodites can also escape the need for a reproductive partner by self-fertilization (which, however, may produce low-fitness offspring due to “inbreeding depression;” see below). This advantage of reproductive assurance is particularly pronounced in sessile animals—like corals—and plants, which cannot move to find a mate [21],[22]. Thus there is a clear advantage to combining both male and female functions within an individual, especially in taxa with low mobility.

However, in some plants and most animals, species are driven to separate the sexes. This can be achieved in several ways. One partial solution is the spatial separation of male and female gonads in the same individual, as in monoecious plants with separate male and female flowers (e.g., maize) and in most hermaphroditic animals. Alternatively, male and female function can be separated in time within an individual, as found in many plants (“dichogamy,” [23]) and some animals (“sequential hermaphroditism,” [24]); slipper shells, for example, are born male and become female later in life. Finally, male and female reproductive organs can be segregated into different individuals, as in some plants (such as papaya and cannabis) and most animals.

Separate sexes have evolved independently many times among plants and animals, which suggests that there must be an evolutionary cost to hermaphroditism, at least in some groups. Two major hypotheses have been proposed to explain the evolution of separate sexes. The first hypothesis is that there are trade-offs between male and female function, such as when mating displays enhance male fitness but decrease female fitness. In this case, individuals can gain reproductive advantages by specializing as a male or female [25]. Direct evidence for the trade-off hypothesis is sparse [26], and observations consistent with it come from hermaphroditic great pond snails, which reallocate resources to female function when sperm production is experimentally abolished [27], and from strawberries, in which increased pollen production comes at the cost of reduced seed set [28]. Indirect evidence of a trade-off comes from the fact that many asexual animals [29] and plants [30] that still have residual sperm/pollen production evolve reduced investment in male gametes over time, suggesting that doing so increases female function. The second major hypothesis is that separate sexes evolve as a means to avoid self-fertilization, which can produce low-fitness offspring because of the exposure of recessive deleterious alleles (“inbreeding depression”) [31]. Empirical evidence for inbreeding depression is widespread in animals and plants [32],[33]; for instance, in the Hawaiian endemic plant genus *Scheidia*, high inbreeding depression promotes the evolution of dioecy [34].

**Figure 2:**
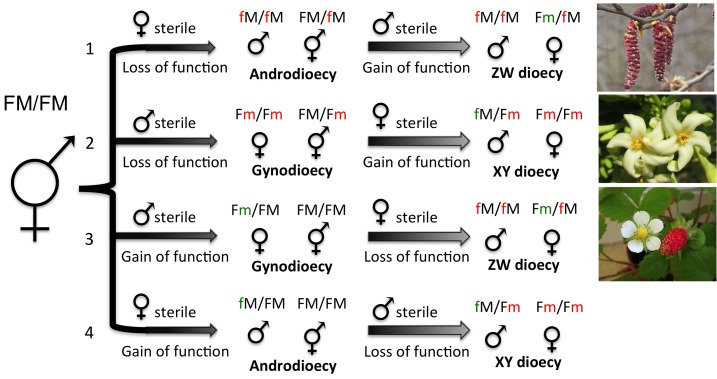
Evolutionary pathways from hermaphroditism to separate sexes. Shown are two-step pathways involving intermediate male- and female-sterile individuals. Loss-of-function mutations (red) are assumed to be recessive, while gain-of-function mutations (green) are assumed to be dominant. Ancestral alleles are in black. M, male fertility allele; m, male sterility mutation; F, female fertility allele; f, female sterility mutation. Because loss of function mutations (red) are almost 50 times more frequent than gain of function mutations (green) in flowering plants, we would expect pathways 1 (e.g., some poplar species) or 2 (e.g., papaya) to arise earlier. Furthermore, transitions through gynodioecy, pathways 2 and 3 (e.g., strawberry) allow females to completely avoid inbreeding depression, while transitions through androdioecy are more costly because males must compete with hermaphrodites for fertilization and do not have any of their own ovules to fertilize. These theoretical arguments help to account for the prevalence of gynodioecy and the XY chromosome system (via pathway 2) observed in plants; nevertheless, all four pathways may be biologically relevant, although no known examples for pathway 4 currently exist.

When separate sexes are favored, the transition can occur via several evolutionary pathways. Separate sexes may evolve from hermaphrodites either by gradual increases in sex-specific investment or rapidly by the appearance of male- or female-sterility mutations (Figure 2). The latter occurs regularly in plants, often generating mixed sexual systems, such as gynodioecy (mixtures of females and hermaphrodites) and androdioecy (mixtures of males and hermaphrodites). Figure 2 highlights the possible pathways for the evolution of separate sexes from a hermaphrodite ancestor and illustrates their relation to sex chromosome evolution. While we have emphasized the evolutionary transition from hermaphroditism to separate sexes, several examples are known where the opposite transitions occur (e.g., [35],[36]), indicating that the conditions favoring the separation of male and female function are not always present.

## Myth 1 Revisited—Sex-Determining Mechanisms Are Diverse and Can Evolve Rapidly

**Figure 3:**
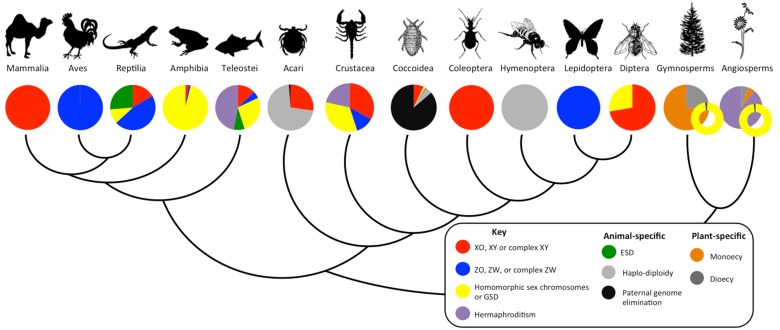
Diversity of sex determination systems for representative plant and animal clades. The bubble insert graph for the plant clades represents the relative proportion of species with documented sex chromosomes within plants with separate sexes. Vertebrates: Mammalia (placental, marsupial, and monotreme mammals), Aves (birds), Reptilia (turtles, snakes, crocodiles, lizards), Amphibia (frogs, toads, salamanders), and Teleostei (bony fishes). Invertebrates: Acari (mites and ticks), Crustacea (shrimps, barnacles, crabs), and Insects, which include Coccoidea (scale insects), Coleoptera (beetles), Hymenoptera (ants, bees, and wasps), Lepidoptera (butterflies), and Diptera (flies). Plants: Gymnosperms (non-flowering plants) and Angiosperms (flowering plants).

**Figure 4:**
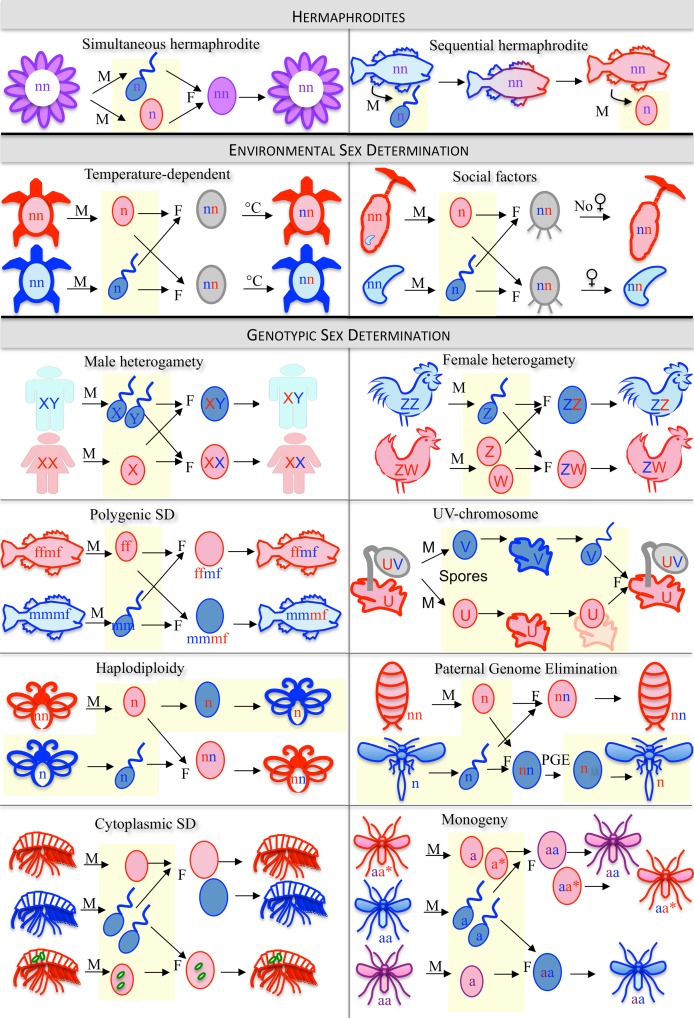
Schematic overview of some sex determination (SD) mechanisms. M refers to meiosis, F to fertilization. Haploid stages (n) are indicated as shaded areas and diploid stages (nn) are unshaded. **Hermaphrodites:** Most flowering plants (and gastropods and earthworms) simultaneously contain both male and female sexual organs (*simultaneous hermaphrodites*). Many fish and some gastropods and plants are *sequential hermaphrodites*; clownfish, for example, are born males and change into females, while many wrasses or gobies begin life as females and then change to males. **Environmental Sex Determination:** In turtles and some other reptiles, sex is determined by incubation temperature of the eggs (*temperature-dependent sex determination*). *Social factors* can act as primary sex-determining cues: sexually undifferentiated larvae of the marine green spoonworm that land on unoccupied sea floor develop into females (and grow up to 15 cm long), while larvae that come into contact with females develop into tiny males (1–3 mm long) that live inside the female. **Genotypic Sex Determination:** Almost all mammals and beetles, many flies and some fish have *male heterogamety* (XY sex chromosomes), while *female heterogamety* (ZW sex chromosomes) occurs in birds, snakes, butterflies, and some fish. In mosses or liverworts, separate sexes are only found in the haploid phase of the life cycle of an individual (*UV sex chromosomes*). In some flowering plants and fish, such as zebrafish, sex is determined by multiple genes (*polygenic sex determination*). In bees, ants, and wasps, males develop from unfertilized haploid eggs, and females from fertilized diploid eggs (*haplodiploidy*), while males of many scale insects inactivate or lose their paternal chromosomes (*paternal genome elimination*). In some species, sex is under the control of cytoplasmic elements, such as intracellular parasites (e.g., *Wolbachia*) in many insects or mitochondria in many flowering plants (*cytoplasmic sex determination*). In some flies and crustaceans, all offspring of a particular individual female are either exclusively male or exclusively female (*monogeny*).

In animals and plants that have evolved separate sexes, accurate differentiation into fertile males and females is a fundamental developmental process. Contrary to Myth 1, however, diverse mechanisms are used to determine sex [3],[4] (Figure 3, Figure 4; Box 2). All crocodiles, most turtles, and some fish exhibit temperature-dependent sex determination; *Wolbachia* infections override existing sex determination systems in many arthropod species and either kill/sterilize males or transform them into phenotypic females; male scale insects eliminate their father's genome after fertilization; marine worm Bonellidae larvae develop as males only if they encounter a female; and many plants and animals—including some snails and fish—change sex during their lifetime in response to environmental or social cues [3],[37].

**Box 2. Glossary of Sex-Determining Mechanisms**

- Hermaphrodites: individuals that contain both male and female sex organs.
- Simultaneous hermaphroditism: male and female sexual organs coexist in one individual (e.g., most flowering plants and earthworms, many terrestrial gastropods).
- Sequential hermaphroditism: individuals change sex at some point during their life (e.g., many fish, snails, and some plants).
- Dioecy (plants) or gonochorism (animals): individuals are either male or female throughout their life.
- Environmental sex determination: sex is triggered by environmental cues, such as temperature, pH, social interactions, and seasonality (e.g., many reptiles and some fish).
- Genotypic sex determination: an individual's sex is established by its genotype (e.g., mammals, birds, amphibians, most insects, some reptiles and fish, and some plants).
- Male heterogamety: type of genotypic sex determination in which males are heterozygous for the sex-determining locus (termed X and Y, as seen in therian mammals and *Drosophila*).
- Female heterogamety: type of genotypic sex determination in which females are heterozygous for the sex-determining locus (termed Z and W, as seen in birds, snakes, butterflies, and gingko trees).
- UV sex determination: separate sexes are only found in the haploid phase of the life cycle of an individual (e.g., mosses or liverworts).
- Polygenic sex determination: sex is determined by multiple genes (e.g., some fish and flowering plants).
- Haplodiploidy: males develop from unfertilized, haploid eggs, and females from fertilized, diploid eggs (e.g., bees, ants, and wasps).
- Paternal genome elimination: paternal chromosomes in males are inactivated or lost after fertilization, leaving males functionally haploid (e.g., many scale insects).
- Cytoplasmic sex determination: sex is under the control of cytoplasmic elements, such as intracellular parasites (e.g., *Wolbachia* in many insects) or mitochondria (e.g., cytoplasmic male sterility in flowering plants).
- Monogeny: all offspring of a particular individual female are either exclusively male or exclusively female (e.g., some flies and crustaceans).
- Sexual reproduction: the mixing of genomes via meiosis and fusion of gametes.
- Sex: the sexual phenotype of an individual.
- Sex determination: the mechanism by which the sexual phenotype of an individual is established in a given species.
- Sex chromosome: a chromosome involved with determining the sex of an individual.
- Autosome: a chromosome not involved with determining the sex of an individual (i.e. any chromosome that is not a sex chromosome).
- Y degeneration: the loss of genetic information on the non-recombining Y chromosome.
- Homomorphic sex chromosomes: sex chromosomes that are morphologically indistinguishable.
- Heteromorphic sex chromosomes: sex chromosomes that are morphologically distinct.
- Sexually antagonistic selection: selection for a trait that benefits one sex to the detriment of the other sex.
- Gynodioecy: a breeding system that consists of a mixture of females and hermaphrodites.
- Androdioecy: a breeding system that consists of a mixture of males and hermaphrodites.
- Meiotic drive (also called segregation distortion): a system in which genetic elements termed segregation distorters bias the proportion of gametes that carry them, resulting in over- or under-representation of one gametic type (i.e. non-mendelian segregation).
- Nucleo-cytoplasmic conflict: conflict in inheritance patterns between the nuclear genome and organelle genomes that are transmitted only maternally.
- Gynandromorphs: individuals that contain both male and female characteristics.

In fact, sex determination is a rapidly evolving trait in many lineages (Figure 3), and sometimes closely related species, or populations of the same species, have different modes of sex determination [3],[4],[38]. Houseflies, for example, normally have XY sex chromosomes, but dominant masculinizing and feminizing alleles on other chromosomes exist in some populations that override sex determination by the XY chromosomes [39]. This variety has stimulated investigation into what evolutionary forces drive the turnover of sex determination mechanisms, what molecular mechanisms underlie the different modes of sex determination, and why sex determination is labile in some taxa and not in others.

### Genotypic versus environmental sex determination

With *genotypic sex determination* (GSD), which occurs in the majority of species with known sex-determining mechanisms, genetic elements specify whether individuals are female or male. In many animals and some plants, however, the switch to develop into a female or male does not lie in the genes. With *environmental sex determination* (ESD), external stimuli control sex determination, such as temperature in reptiles [40], photoperiod in marine amphipods and some barnacles [41],[42], and social factors in many coral-reef-dwelling fish and limpets [43],[44]. Exactly how the environment triggers sex development has remained an open question, although a recent study found that methylation provided the link in European sea bass [45]. In many species, the line between GSD and ESD is blurred, with certain environments altering the (otherwise genetically determined) sex of developing offspring [46]. For example, tongue sole have differentiated ZW sex chromosomes, but ZW embryos develop into males when incubated at high temperatures, and sex reversal is accompanied with substantial methylation modification of genes in the sex determination pathway [47].

ESD is favored over GSD when specific environments are more beneficial to one sex [3], selecting for sex-determining mechanisms that match each sex to its best environment. For example, in some gobies and wrasses, nest sites are limited, and a male's ability to defend his nest depends on body size; individuals tend to start life as females, and only become males once they are sufficiently large to successfully defend a nesting site [48]. The reverse transition, from ESD to GSD, is thought to be favored when the environment is unpredictable or not variable enough, in which case ESD could produce strongly skewed sex ratios or intersex individuals [3]. Indeed, snow skinks, which are small, live-bearing lizards, have different sex-determining mechanisms in different environments. ESD occurs at low altitudes where early birth is advantageous for females and the variance in temperature between years is low. GSD predominates at high altitudes where there is no advantage for early-born females and between-year variance in temperature is high [49]. In this situation, ESD produces optimal sex ratios at low elevations, while GSD prevents extreme sex ratios at high altitudes. Importantly, global climate change poses a threat to species with temperature-dependent sex determination if they cannot adapt rapidly enough to avoid biased sex ratios [50]. Another threat to ESD systems comes from within: they may be prone to invasion by genetic elements that favor biased sex ratios (see below).

### Genomic conflict and transitions in sex determination

More generally, selection on the sex ratio can trigger transitions between and among different ESD and GSD systems [3]. Sex-biased inheritance patterns of different genetic elements—such as sex chromosomes, organelles, or endosymbionts—create a conflict over which sex is preferred, and can drive the evolution of male- or female-biased sex ratios. In populations with a skewed sex ratio, selection on autosomal genes typically favors equal investment in males and females [51],[52], and a new GSD or ESD system can become established if it restores a more even sex ratio. An equal number of males and females is, however, not always favored, even among autosomal genes (e.g., with local mate competition, [53]). In this case, selection for biased sex ratios can favor the establishment of a new sex-determining system [54].

Many examples are known of sex chromosomes, organelles, and endosymbionts that bias the primary sex ratio. Meiotic drive, where genetic elements bias the proportion of gametes that carry them, can create male-biased sex ratios if they are located on the Y or Z chromosomes (as seen in many *Drosophila* species [55]), whereas driving X or W chromosomes create female-biased sex ratios (found in *D. simulans* [56], stalk-eyed flies [57], and rodents [58]); autosomal genes that restore unbiased sex ratios are known in many systems. Cyto-nuclear conflict arises because cytoplasmic factors such as mitochondria or chloroplast are often inherited only through the mother, and they favor production of females, while autosomal genes are inherited through both sexes and favor more equal sex ratios. Cytoplasmic male sterility encoded by mitochondria has been widely reported in plants, including maize, petunia, rice, common bean, and sunflower [59], as have nuclear-encoded male fertility restorer genes [60]. Likewise, cellular endosymbionts are only transmitted through the mother and can create maternally inherited female-biased sex ratios; examples include male-killing bacteria in butterflies and *Drosophila* [61],[62]. Recurrent invasions of sex ratio distorters and their suppressors can result in rapid transitions among sex determination mechanisms between species, and may be a major force contributing to the diversity of sex-determining mechanisms observed across the tree of life.

### Turnover of sex chromosomes

**Figure 5:**
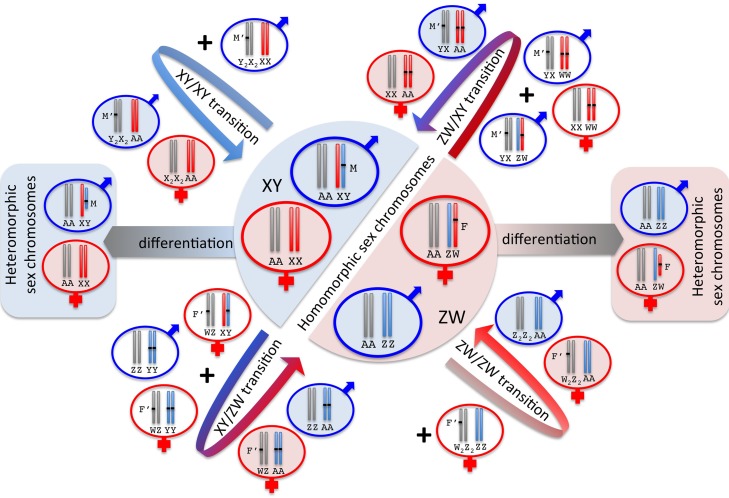
Transitions versus differentiation of sex chromosomes. Transitions between homomorphic sex chromosomes result from new masculinizing (M′) or feminizing (F′) mutations that invade an existing XY or ZW system to create a new chromosome pair (in grey) that harbors the sex-determining gene (additional transitional karyotypes are indicated by unshaded circles). XY→XY transitions result in the loss of the ancestral Y (and ZW→ZW transitions cause loss of the ancestral W). Transitions between XY and ZW systems result in some offspring that are homozygous for the Y (blue) or W (red) chromosome and are thus more likely if the chromosomes have similar gene content but become increasingly difficult if these chromosomes have degenerated (side boxes on left and right), causing YY and WW individuals to be less fit.

In species with genotypic sex determination, the chromosome pair that determines sex can change rapidly over time. Transitions are particularly likely when the ancestral sex chromosome exhibits little genetic differentiation, since WW or YY combinations are then less likely to be lethal (Figure 5). New sex-determining genes (or copies of the original gene in a new location) can lead to transitions within and between different XY and ZW systems (Figure 5). Invasions of sex-determining genes are facilitated when the new sex-determining gene (or a gene closely linked to it) has beneficial effects on fitness [63].

Sexually antagonistic selection, which occurs when a mutation is beneficial to one sex but detrimental to the other, can also drive transitions between sex determination by different pairs of chromosomes [64],[65]. For example, if an allele of an autosomal gene is beneficial to males but harmful to females and becomes linked to a dominant masculinizing mutation, then chromosomes that carry both the male-beneficial and male-dominant alleles create a novel Y that can replace the ancestral mechanisms. Conversely, alleles that benefit females and harm males can create novel W chromosomes when linked to feminizing mutations. Turnover of sex chromosomes can also be triggered by the degeneration of the Y and W chromosome, which commonly follows the cessation of recombination [66],[67], and will result in the replacement of a low-fitness Y or W chromosome with a nondegenerate one [68].

### Sex determination by the whole genome

In many animals, sex determination involves the entire genome. With haplodiploidy (found in about 12% of animal species, including all ants, wasps, and bees) and paternal genome elimination (found in scale insects), males only transmit their maternal set of genes (see Figure 4; Box 2: Glossary). The loss of the paternal genome in sons benefits mothers but not fathers because these uniparental sons transmit more of a mother's genome to grandchildren than do biparental sons [3]. Females also experience a selective advantage from haplodiploidy (but not paternal genome elimination) because unfertilized eggs can develop and contribute to fitness when mating opportunities are rare.

Despite numerous theoretical predictions for how and why sex determination mechanisms change, many hypotheses remain untested. Only a small proportion of taxa have actually been characterized for their sex determination mechanisms, hindering the use of comparative methods to assess the factors associated with transitions between them. However, because sex determination changes so rapidly in many clades, we can catch these transitions *in action* to test theoretical predictions in a direct, experimental way.

## Myth 2 Revisited—Multiple and Various Genes Can Determine Sex

The pathways that control sexual development have been well characterized at the molecular level in *D. melanogaster*, *C. elegans*, and mammals. All three involve a master-switch sex-determining gene, which led to the birth of Myth 2. Although the simplicity of a single master-switch is alluring, this archetype of sex determination is clearly not universal. Below we discuss systems where sex is determined by multiple genes, recent molecular data on the nature and evolution of sex-determining genes, and how sex determination can vary in different parts of the body.

### Polygenic sex determination

In some species, sex determination is polygenic. For example, in zebrafish (*Danio rerio*), a key developmental model organism, sex is not controlled by a single master regulator but is instead a quantitative threshold trait with either a male or female outcome, which is determined by multiple regions in the genome [69]–[71]. While some of those regions contain genes known to play a role in sex determination in other organisms [70], there is an enduring mystery as to how these multiple loci and the environment interact to control downstream sexual differentiation in zebrafish. Zebrafish gonads develop as testes in the absence of signals from germ line cells, suggesting that the factors determining sex may regulate germ cell proliferation [72]. Sex as a threshold trait has been inferred in several other fish [73]–[75] and invertebrates [76], and further examples of multiple interacting loci controlling sex determination are no doubt waiting to be described. Indeed, in taxa where separate sexes evolved recently from a hermaphrodite ancestor, as is common in plants, multiple sex-determining loci are in fact expected, since at least two independent mutations—one suppressing male function, one suppressing female function—are necessary to produce separate sexes from a hermaphrodite (Figure 2). If separate sexes evolve by gradual increase in sexual investment from a hermaphrodite, sex determination may also be due to polygenic inheritance.

### The nature and evolution of sex-determining genes and pathways

Some taxa have master-switch sex-determining genes that are highly conserved, such as the *Sry* gene in nearly all mammals [77]. In other lineages, such as fish from the genus *Oryzias* [78]–[80], the master-switch gene differs among closely-related species (Table 1). There is some empirical evidence for the repeated use of the same master sex determination switch genes in animals. For example, in vertebrates other than mammals, *dmrt1* (a DM family gene) and its paralogs act as the primary sex determination signal in African clawed frog (*Xenopus laevis*) [13], chicken (*Gallus gallus*) [12], medaka fish (*Oryzias latipes*) [78],[79], and possibly the smooth tongue sole (*Cynoglossus semilaevis*) [14]. In insects, paralogs of *transformer* (*tra*), a key gene in the sex determination cascade of *Drosophila*, have evolved as the primary switch in houseflies *Musca domestica* [17], as well as the haplodiploid wasp *Nasonia vitripennis* [15] and the honeybee *Apis mellifera* [16].

**Table 1:** Known master sex-determining genes in vertebrates and insects, and their paralogs.

Species	Master sex determining gene	Sex-determining mechanisms	Gene paralog	Paralog function	Reference
mammals	*Sry*	sex-determining Y	*Sox3*	HMG-box transcription factor	[77]
chicken (*Gallus gallus*)	*dmrt1*	dose-dependent Z	*-*	SD pathway transcription factor	[12]
African clawed frog (*Xenopus laevis*)	*dmW*	sex-determining W	*dmrt1*	SD pathway transcription factor	[13]
medaka (*Oryzias latipes*)	*dmrt1Y*	sex-determining Y	*dmrt1*	SD pathway transcription factor	[78],[79]
(*Oryzias luzonensis*)	*gsdfY*	sex-determining Y	*gsdf*	secretory protein in SD pathway	[80]
Patagonian pejerrey (*Odontesthes hatcheri*)	*amhY*	sex-determining Y	*amh*	anti-Mullerian hormone	[155]
rainbow trout (*Oncorhynchus mykiss*)	*sdY*	sex-determining Y	*Irf9*	interferon regulatory factor	[82]
tiger pufferfish (*Takifugu rubripes*)	*amhr2*	dose-dependent X	*amhr*	anti-Mullerian hormone receptor	[156]
smooth tongue sole (*Cynoglossus semilaevis*)	*dmrt1*	dose-dependent Z	*-*	SD pathway	[14]
fruit flies (*Drosophila*)	*Sxl*	dose-dependent X	*CG3056*	mRNA splicing, non-sex specific	[83],[84]
housefly (*Musca domestica*)	*F*	sex-determining W	*tra*	SD pathway switch splice factor	[17]
silkworm (*Bombyx mori*)	*Fem*	sex-determining W	*-*	piRNA	[85]
honeybee (*Apis mellifera*)	*csd*	haplodiploid	*tra*	SD pathway switch splice factor	[16]
wasp (*Nasonia vitripennis*)	*Nvtra*	haplodiploid	*tra*	SD pathway switch splice factor	[15]

These data suggest that there are constraints on the types of genes that can be co-opted as master sex determination genes [81]. Nevertheless, there are several cases of switch genes with no homologs in closely related taxa. These include an immunity-related gene in rainbow trout (*Oncorhynchus mykiss*) [82] and *Sxl* in *Drosophila* [83], whose ortholog has a non-sex-related function in mRNA splicing in other flies [84]. The primary master sex-determining gene in the silkworm *Bombyx mori* is a W-derived female-specific piRNA (produced from a piRNA precursor termed *Fem*) that targets a Z-linked gene (named *Masc*), and silencing of *Masc* mRNA by *Fem* piRNA is required for female development [85]. Undoubtedly, many other sex determination genes remain to be found, making it unclear at present whether there truly are constraints on the types of genes that could evolve to be master control switches.

No master sex determination gene has been identified in dioecious plants, but genes that affect flower sex determination have been found [86],[87]. Indeed, many genes may serve as potential targets for sex determination in plants, given that male or female sterility can evolve in various ways [86]. For example, 227 male-sterility genes have been identified in rice, with at least one male-sterility gene found on each of rice's 12 chromosomes—hence each autosome could, in principle, evolve a sex-determining function [88]. This abundance and diversity within a single species indicates that the initial evolution of separate sexes is unlikely to be limited to a scant handful of master genes.

In sharp contrast with the diversity of primary sex-determining signals, some key regulatory genes play conserved roles in the molecular pathways leading to male or female gonad development across invertebrates and vertebrates, such as the *doublesex-mab3* (DM) family genes [89],[90]. Despite profound differences in the mode of sex determination and the identity of the master-switch genes, DM genes are specifically expressed in the developing gonads of almost all animals, including vertebrates (mammals [91], birds [92], turtles and alligators [93]–[95], amphibians [96], and fish [97]) and invertebrates (*Drosophila* [98], hymenoptera [99], crustaceans [100],[101], and mollusks [102],[103]). Thus, the evolution of sex-determining pathways, at least in animals, appears to occur by the recruitment of new master-switches controlling sexual fate, while the downstream developmental pathways that regulate gonadal differentiation are retained [10],[81],[104], although the function of some of these downstream elements appears to diverge among lineages [105]. Characterization of polygenic sex determination systems and identification of master sex determination genes across kingdoms will provide insight into the mechanistic constraints limiting the evolution of sex determination pathways.

### Sex determination: soma vs. germ line

Sex determination can also differ with respect to where in the body sex is determined. In humans, sex is determined in the developing gonad, and gonadal sex hormones in turn trigger sex determination and differentiation in nongonadal tissues. By contrast, in birds, *Drosophila*, and nematodes [106]–[109], sexual differentiation is a cell-autonomous process, although secreted signaling molecules can play a role in generating sexual dimorphism in somatic tissues. Studies in *Drosophila* have shown that only a subset of cells express the genes of the sex determination cascade and have a sexual identity [106]. Cell-autonomous sex determination can result in the formation of gynandromorphs—individuals that contain both male and female characteristics, found in birds and many insects, including butterflies and beetles. Sex determination can also be regulated differently in the soma versus the germ line of the same species [110],[111]. In houseflies [112] and some frogs [113] and fish [114]–[116], transplantation experiments indicate that the sex of germ cells depends on the surrounding soma, i.e., XX germ cells transplanted into male soma form sperm, and XY germ cells transplanted in a female soma form oocytes. In contrast, germ cells in *Drosophila* [117] and mammals [118] receive signals from the surrounding somatic gonad, but they also make an autonomous decision during germ line sexual development; this may also be true for chickens [107]. In these animals, the “sex” of the soma must match the “sex” of the germ cells for proper gametogenesis to occur. If XX germ cells are transplanted into male soma they do not form sperm, and XY germ cells transplanted into female soma fail to form oocytes.

## Myth 3 Revisited—Sex Chromosomes' Eternal Youth

Heteromorphic sex chromosomes evolve from autosomes that are initially identical but then stop recombining and differentiate. Recombination suppression is favored when it links together sexually antagonistic alleles and sex-determining loci (i.e., a male-beneficial allele and a male-determining gene on a Y chromosome, or a female-beneficial allele and a female-determining gene on a W chromosome). A side effect of repressed recombination on Y and W chromosomes is that natural selection is inefficient (reviewed in [4],[5]), which can result in the loss of most of their genes. Y or W degeneration has occurred in many animal taxa with heteromorphic sex chromosomes, including mammals [119], many birds [120], snakes [121], and many insects [122],[123], along with some plants, including *Rumex* [124]. In the most extreme cases, the Y or W is entirely lost, resulting in so-called X0 and Z0 systems. According to Myth 3, differentiation of sex chromosomes is evolutionarily inevitable, and the degree of heteromorphism reflects their age (Figure 5). However, as we explain below, evidence from a broad array of organisms indicates that the link between sex chromosome heteromorphism and age is often far from direct.

### Not all sex chromosomes become differentiated

Differentiation is often seen as the default path of sex chromosome evolution, but contrary to Myth 3, some ancient sex chromosomes recombine and are undifferentiated over most of their length. Examples are found in python snakes and ratite birds, whose homomorphic sex chromosomes are about 140 and 120 million years old, respectively [121],[125],[126], i.e. almost as old as the heteromorphic sex chromosomes of therian mammals (about 180 million years old).

How do some ancient sex chromosomes avoid differentiation? One hypothesis is that occasional X-Y recombination purges deleterious alleles on the Y. A mechanism proposed for tree frogs is that XY embryos are occasionally sex-reversed, and so the X and Y recombine when these embryos develop into females [127],[128]. Second, some taxa may have few genes under sexually antagonistic selection on their sex chromosomes and thus avoid selection to suppress recombination between the X and Y [129]. Third, sexually antagonistic selection can be resolved by other means, such as the evolution of sex-specific expression [130]. Sexually antagonistic alleles can accumulate along the sex chromosomes, and sex-specific expression will confine the product of such alleles to the sex they benefit, thereby eliminating the selective pressure for recombination suppression. Consistent with this last possibility, the recombining, non-differentiated region along the sex chromosomes of the emu (a ratite bird) contains an excess of genes whose expression is sex-biased, relative to autosomes [126].

### Y chromosomes are not doomed

Y chromosome degeneration has prompted the suggestion that the human Y will eventually disappear [131]–[133], a claim based on the naïve assumption of a constant rate of gene loss from the Y over time. However, theory predicts that the rate of gene decay on the Y decreases over evolutionary time and should halt on an old, gene-poor Y chromosome [67],[134]. Recent comparative genomic studies support this hypothesis as the gene content of the primate Y chromosome has been stable over the last 25 million years, suggesting that an equilibrium gene content has been reached in humans [135]. Moreover, old gene-poor Y chromosomes that are tens of millions of years old, such as the *Drosophila* Y [136], actually show a net rate of gene gain rather than gene loss [137]. Thus, the Y chromosome can be a stable and important component of the genome in many species, and may even prevent turnover of sex-determining mechanisms (see below).

### Evolutionary traps and conserved sex-determining systems

In contrast to the lability of sex determination mechanisms in some groups, eutherian mammals, birds and many insects exhibit virtually no variation in how sex is determined (Figure 3). This stability could be due to an absence of genetic variation, particularly when multiple genetic steps are required for a transition to a new sex-determining system (Figure 2). Mutations are known, however, that override sex determination (Table 1) [138], suggesting that the conservation is not due to a lack of genetic variation. Instead, evolutionary traps may stabilize sex-determining systems for long spans of evolutionary time.

Heteromorphic sex chromosomes may act as just such a trap. Transitions between XY and ZW systems that create YY or WW individuals are prevented when Y or W chromosomes lack essential genes (Figure 5). Also, if the Y (or W) chromosome has evolved sex-essential genes, such as spermatogenesis genes located on the human and *Drosophila* Y, sex chromosome transitions are only possible if these genes are moved to another chromosome, since the old Y, along with its genes, is lost during the transition (Figure 5). Overall, phylogenetic patterns in vertebrates or insects [3],[139] are consistent with the notion that heteromorphic sex chromosomes constrain shifts in sex determination mechanism, but several notable exceptions exist in both groups. In rodents, for example, many species with unusual sex-determining systems can be found: XY females in some lemming species, X0 females or XX males in vole species, and X0 females and males in some Japanese spiny rats and mole voles [140]. Likewise, some insect groups are known that harbor variation in sex chromosome karyotype among species; in grasshoppers, fusions between sex-chromosomes and autosomes combined with Y-degeneration and/or Y-loss have created much variation in sex chromosome karyotype, including species with multiple X or Y chromosomes [141]; true fruit flies (Tephritidae) that contain both XY and ZW species [142]; or blowfly species that have secondarily lost their heteromorphic sex chromosomes [143].

How much sex chromosome heteromorphism is required to create a trap, and how strong this trap is, remains unknown. To date, only one example of the reversal of an ancient sex chromosome back to an autosome has been characterized. Specifically, all *Drosophila* species contain an autosome that was formerly an X chromosome: the dot chromosome. This chromosome still has a minor feminizing role during sex determination, is targeted by a chromosome-specific regulatory mechanism similar to dosage compensation of the X, and its patterns of biased gene expression during early embryogenesis, oogenesis, and spermatogenesis resemble that of the current X in *Drosophila* [136]. The retention of the specialized genomic architecture of highly differentiated sex chromosomes on the dot chromosome illustrates the numerous barriers to sex chromosome turnover that exist in highly heteromorphic systems, even though there are some cases where these are overcome.

Haplodiploidy also appears to be an evolutionary trap. While it has arisen a few dozen times, the reverse transition has not been reported [3]. Transitions from or to haplodiploidy require changes in genetic architecture and meiotic mechanisms, which are likely more complex than a simple change in a master-switch sex-determining gene. Furthermore, females switching from haplodiploidy would lose the fitness benefit associated with producing uniparental sons.

Systems that involve interacting somatic and germ line sex determination mechanisms may also limit transitions of sex-determining mechanisms, since changes in either germ line sex or somatic sex alone may produce infertile individuals [111]. Thus, while sex determination is generally characterized by diversity and turnover, some sex-determining systems appear to be more evolutionarily stable than others [3].

## Outlook

Studying the forces that drive the evolution of sex determination has mainly come from theoretical works, with little empirical data. However, the genomic revolution has allowed researchers to address scientific questions and tackle novel biological systems at the molecular level. As new genomic approaches increase the pace of discovery and characterization of sex determination innon-model organisms, we anticipate that comparative phylogenetic methods will be key to examining the roles of various ecological and genetic factors that drive changes in sex determination mechanisms. Additionally, genomic data make it increasingly possible to map sex-determining loci from closely related species and to identify the evolutionary mechanisms hypothesized to cause transitions among sex-determining systems. Finally, comparative and functional genomic data will allow researchers to address how new master sex determination genes are incorporated into existing genetic networks controlling sexual development. A full understanding of the diversity of sex determination mechanisms will require that we expand the taxonomic breadth of study systems well beyond classic model organisms. Promising models include dipteran insects, such as houseflies or chironomids; teleost fish; and reptilian clades, including turtles and lizards; as well as plant genera, such as strawberries, that show variation within and between species in how sex (or gender in plants) is determined. Integrative and interdisciplinary approaches across the tree of life will illuminate the diversity of sex determination and yield exciting new insights of how and why sex determination evolves in animals and plants.
